# GPs should not try to detect mild COPD

**DOI:** 10.1038/s41533-020-0176-0

**Published:** 2020-05-11

**Authors:** Paul Enright, Carlos Vaz Fragoso

**Affiliations:** 1 Department of Medicine, the University of Arizona, Tucson, AZ USA; 2 Clinical Epidemiology Research Center, VA Connecticut Healthcare System, Mailcode 151B, West Haven, CT USA

**Keywords:** Chronic obstructive pulmonary disease, Respiratory signs and symptoms

More than 40 years ago, Robert Hyatt and Paul Enright at The Mayo Clinic in Minnesota wrote an office spirometry book in which primary care practitioners were encouraged to detect COPD early^1^, and concluded: “Spirometry should be performed for all patients over the age of 40 years who smoke cigarettes [but] we do not advocate mass screening”. More than 10 years ago, Enright wrote an editorial for this journal about the use and abuse of office spirometry, concluding that a normal peak expiratory flow (PEF) rules out clinically important COPD, and for smokers with a low PEF, before prescribing expensive daily inhalers, GPs should refer the patient to a local service for good-quality pre- and post-bronchodilator (BD) spirometry and interpretation^2^. Here, we review studies about detecting mild COPD published since that editorial.

First, the rates of COPD overdiagnosis are now unacceptably high, causing many patients to be taking a daily inhaler that they do not need. A 2019 review of 28 studies reporting rates of COPD overdiagnosis and misdiagnosis^3^ found the following causes: (1) physicians did not use post-BD spirometry to establish the diagnosis; (2) the results were incorrectly interpreted; (3) co-morbidities with similar symptoms and abnormal spirometry (such as asthma or heart failure) were not considered; (4) spirometry was normal during a follow-up exam. For example, 31% of over 6000 patients admitted to a community teaching hospital with COPD as the leading diagnosis had normal spirometry results during their hospital stay^4^. One-third of 187 patients with dyspnea who had been previously diagnosed with both chronic heart failure and COPD did not have airway obstruction^5^. In another study of smokers with respiratory symptoms and a diagnosis of COPD, over 85% did not have post-BD airway obstruction^6^. All guidelines agree that a single spirometry test without airflow obstruction (the “O” in COPD) rules out COPD for that patient.

Since 2001, the industry-sponsored GOLD guidelines for COPD have inappropriately defined “mild” and “moderate” COPD^7^. The vast majority of smokers with a low FEV1/FVC ratio but a normal FEV1, are labeled as having mild COPD (the old GOLD stage 1). But according to the large COPDGene study (>10,000 smokers, ages 45–81), they have normal pulmonary phenotypes: dyspnea grade, SGRQ, 6-min walking distance, bronchodilator reversibility, and lung CT scans (% emphysema, % gas trapping, and small airway dimensions)^8^. In other words, their respiratory symptoms are not due to mild COPD, and a search for other causes of their respiratory symptoms should be undertaken. Those who are smokers must be encouraged and helped to quit smoking, and inhalers are not indicated unless they have asthma.

Josephs et al. identified over 14,000 patients with a COPD diagnosis from a large UK database^9^ and reviewed all of their prescriptions and spirometry test results. During their median COPD duration of 5 years, about half had persistent airflow obstruction (AFO), i.e., low FEV1/FVC at all visits, one-third had intermittent AFO, 11% never had AFO (*n* = 1434), and 13% never had a spirometry test. Despite absent AFO, more than half of the 1434 patients were taking a LABA, LAMA, ICS, or combination inhaler every day, including those without a history of asthma. The authors concluded that “patients without AFO require clinical reassessment, as they may be receiving inappropriate, potentially harmful, and costly medications that may not benefit them, while the true cause of their symptoms may have been missed”.

Another UK audit of medical records included 48,105 patients with a diagnosis of COPD^10^. Only 19% had post-BD spirometry. Of these, 25% (*n* = 2255) did not have AFO. Patients with spirometry incompatible with their COPD diagnosis were more likely female, never-smokers, more obese, and have a higher FEV1. In a population-based sample of adults from 20 countries, more than half of those reporting a physician’s diagnosis of COPD had entirely normal spirometry, and over half of them reported current use of a respiratory medication^11^.

Second, the opportunity to diagnose other causes of chronic cough, such as rhinosinusitis, asthma, GERD, and bronchiectasis, is missed. Also, dyspnea due to heart failure is missed. All these diseases have very effective treatments. A Mayo Clinic review of the 2018 GOLD guidelines concluded that COPD “is commonly both overdiagnosed and underdiagnosed because of lack of spirometry testing among symptomatic patients”. This results in inappropriate therapy for many patients and delayed diagnosis of other treatable conditions^12^. Some of the final diagnoses can be ischemic heart disease, congestive heart failure, subglottic stenosis, and pulmonary hypertension, all of which are serious and could lead to patient harm, if unrecognized^13^. “In addition to this risk, patients are often on long-term inhaled therapy unnecessarily, leading both to potential side-effects and significant ongoing healthcare costs, as these drugs are likely to be issued for many years after the misdiagnosis. If the wrong diagnosis is made, patients are also likely to remain symptomatic, and potentially have their treatment stepped up, adding to both the cost and potential for side-effects. At a societal level, overdiagnosis of asthma may lead to significant opportunity cost, as resources required elsewhere are inappropriately spent on over-diagnosed asthma”^14^. Although this author was talking about asthma, the same is true of the misdiagnosis of COPD.

Third, “pre-COPD”, “mild” COPD, and “early” COPD are not diseases, and do not need detection or treatment because no combination of inhaled drugs slows the progression of COPD in a continuing smoker. Clinically important COPD takes decades to develop in susceptible smokers. During this time, large groups of these susceptible smokers may statistically significantly differ (*p* < 0.05) in some respect from the larger group of smokers who will not develop COPD. For example, many types of pulmonary function tests (body plethysmography, diffusing capacity (DLCO), forced oscillation, etc.) and cardiopulmonary exercise tests will become borderline abnormal in susceptible smokers before AFO is detected by spirometry^15–20^. However, these test results are highly unreliable for predicting progression to clinically important COPD^21,22^.

Emphysema can be detected using advanced processing of thoracic CT scans in smokers with normal spirometry. The COPDGene study and others showed that most GOLD stage 1 “mild COPD” smokers had evidence of emphysema (>5%) or airway thickening on quantitative CT-scan analysis^23,24^. However, the positive predictive power of these tests for clinically important COPD is poor. Age-specific reference equations for % emphysema and % gas trapping as determined in healthy populations of asymptomatic lifelong nonsmokers are unavailable^25^. Thus, emphysema seen on lung CT scans may be “senile emphysema” due to normal aging^26^. CT-measured emphysema without AFO was associated with all-cause mortality in a very large study^27^. However, participants who were characterized as “without airflow obstruction” included those with restriction (a reduced FVC but normal FEV1/FVC), which is often due to heart failure or sarcopenia^28^. Borderline airway obstruction has been associated with higher respiratory morbidity during long-term follow-up; however, both asthma and COPD could have caused the morbidity^29–31^.

Many pulmonary physicians (most of whom have industry funding) are optimistic that the early detection of mild COPD will become important^20,32^. However, a recent review of mild COPD admitted that: “Future research should address two major issues: first, whether mild airflow limitation represents an early stage of COPD and … whether regular treatment should be considered for COPD patients with mild airflow limitation…”^33^.

Finally, aging-related concerns further inform the low clinical value of establishing a diagnosis of mild COPD. Ultimately, it is the rapid aging of populations worldwide (1.5 billion persons will be aged ≥65 years by 2050)^34^ that highlights the low clinical value of mild COPD. Older persons have a high burden of respiratory symptoms that are often multifactorial, due to age-related increases in multimorbidity (62% of Americans aged ≥65 years have two or more chronic conditions), medication-related adverse effects, and high rates of severe deconditioning^35^. In such a clinical setting, even with age-appropriate spirometric criteria, mild COPD is only modestly associated with respiratory symptoms, and is otherwise not associated with poor exercise performance^36^. Mild COPD is defined by the World Health Organization as a patient who has a chronic cough or dyspnea, but is able to walk long distances and climb stairs. It has a very low disability weight (lower than mild diarrhea)^37^. Hence, ascribing disability to mild COPD (and attempting to treat it) is clinically inappropriate, especially in older patients.

In summary, we recommend that primary care practitioners (i) do not diagnose or treat COPD until two or more spirometry tests demonstrate moderate-to-severe airway obstruction; (ii) rule out COPD with a normal peak flow^38,39^; (iii) refer patients with respiratory symptoms to a facility where good-quality spirometry is done. Also, it has been said that “everything is “COPD” until the correct diagnosis is made,” so carefully consider causes of a chronic cough or dyspnea other than COPD, even in elderly smokers with abnormal spirometry. Finally, for dyspneic patients with spirometric restriction (a low FVC), we recommend obtaining a chest X-ray (or lung CT scan), a DLCO test, and B-naturetic protein test.

## References

[1] Enright, P. L. & Hyatt, R. E. *Office Spirometry, A Practical Guide to the Selection and Use of Spirometers* (Lea and Febiger, Philadelphia, 1987).

[2] Enright P (2008). The use and abuse of office spirometry. Prim. Care Respir. J..

[3] Thomas ET, Glasziou P, Dobler CC (2019). Use of the terms “overdiagnosis” and “misdiagnosis” in the COPD literature: a rapid review. Breathe.

[4] Spero K, Bayasi G, Beaudry L, Barber KR, Khorfan F (2017). Overdiagnosis of COPD in hospitalized patients. Int J. Chron. Obstruct Pulmon Dis..

[5] Minasian AG (2013). COPD in chronic heart failure: less common than previously thought?. Heart Lung.

[6] Heffler E (2018). Misdiagnosis of asthma and COPD and underuse of spirometry in primary care unselected patients. Respir. Med..

[7] GOLD. *Global Strategy for the Diagnosis, Management and Prevention of COPD, Global Initiative for Chronic Obstructive Lung Disease (GOLD)*. https://goldcopd.org/ (2018).

[8] Vaz Fragoso CA (2015). Phenotype of normal spirometry in an aging population. Am. J. Respir. Crit. Care Med..

[9] Josephs L, Culliford D, Johnson M, Thomas M (2019). COPD overdiagnosis in primary care: a UK observational study of consistency of airflow obstruction. NPJ Prim. Care Respir. Med..

[10] Fisk M (2019). Inaccurate diagnosis of COPD: the Welsh National COPD Audit. Br. J. Gen. Pr..

[11] Sator L (2019). Overdiagnosis of COPD in subjects with unobstructed spirometry: a BOLD analysis. Chest.

[12] Mirza S, Clay RD, Koslow MA, Scanlon PD (2018). COPD guidelines: a review of the 2018 GOLD report. Mayo Clin. Proc..

[13] Aaron SD (2017). Diagnostic instability and reversals of COPD diagnosis in individuals with mild to moderate airflow obstruction. Am. J. Respir. Crit. Care Med..

[14] Kavanagh J, Jackson DJ, Kent BD (2019). Over- and under-diagnosis in asthma. Breathe.

[15] Frantz S (2012). Impulse oscillometry may be of value in detecting early manifestations of COPD. Respir. Med..

[16] Elbehairy AF, Guenette JA, Faisal A (2016). Mechanisms of exertional dyspnoea in symptomatic smokers without COPD. Eur. Respir. J..

[17] Harvey BG (2015). Risk of COPD with obstruction in active smokers with normal spirometry and reduced diffusion capacity. Eur. Respir. J..

[18] Guenette JA (2014). Mechanisms of exercise intolerance in global initiative for COPD grade 1 COPD. Eur. Respir. J..

[19] O’Donnell DE, Laveneziana P, Webb K, Neder JA (2014). COPD: clinical integrative physiology. Clin. Chest Med..

[20] Hoesterey D (2019). Spirometric indices of early airflow impairment in individuals at risk of developing COPD: spirometry beyond FEV_1_/FVC. Respir. Med..

[21] Vaz Fragoso CA, Concato J, McAvay G, Van Ness PH, Gill TM (2012). Respiratory impairment and COPD hospitalisation in older persons: a competing risk analysis. Eur. Respir. J..

[22] Vaz Fragoso CA (2013). Respiratory impairment in older persons: when less means more. Am. J. Med..

[23] Regan EA (2015). Clinical and radiologic disease in smokers with normal spirometry. J. Am. Med. Assoc..

[24] Pirozzi CS (2018). Heterogeneous burden of lung disease in smokers with borderline airflow obstruction. Respir. Res..

[25] Smith BM, Barr RG (2013). Establishing normal reference values in quantitative computed tomography of emphysema. J. Thorac. Imaging.

[26] Vaz Fragoso CA (2016). Epidemiology of COPD in aging populations. COPD.

[27] Oelsner EC (2014). Association between emphysema-like lung on cardiac computed tomography and mortality in persons without airflow obstruction: a cohort study. Ann. Intern. Med..

[28] Vaz Fragoso CA (2017). Spirometry, static lung volumes, and diffusing capacity. Respir. Care..

[29] Park HJ (2018). Significant predictors of medically diagnosed COPD in patients with preserved ratio impaired spirometry: a 3-year cohort study. Respir. Res..

[30] Bhatt SP (2019). Discriminative accuracy of FEV1:FVC thresholds for COPD-related hospitalization and mortality. J. Am. Med. Assoc..

[31] Scichilone N (2015). Choosing wisely: practical considerations on treatment efficacy and safety of asthma in the elderly. Clin. Mol. Allergy.: CMA.

[32] Rennard SI, Drummond MB (2015). Early chronic obstructive pulmonary disease: definition, assessment, and prevention. Lancet.

[33] Rossi Andrea (2017). Chronic obstructive pulmonary disease with mild airflow limitation: current knowledge and proposal for future research - a consensus document from six scientific societies. Int. J. COPD.

[34] World Health Organization. *Global Health and Aging. National Institute on Aging, National Institutes of Health*, No. 11-7737 (NIH Publication, 2011).

[35] Mangin D (2018). International group for reducing inappropriate medication use & polypharmacy (IGRIMUP): position statement and 10 recommendations for action. Drugs Aging.

[36] Vaz Fragoso CA (2016). Phenotype of spirometric impairment in an aging population. Am. J. Respir. Crit. Care Med..

[37] Salomon JA (2015). Disability weights for the global burden of disease 2013 study. Lancet Glob. Health.

[38] Jithoo A (2013). Case-finding options for COPD: results from the burden of obstructive lung disease study. Eur. Respir. J..

[39] Thorat Y, Salvi S, Kodgule R (2017). Peak flow meter with a questionnaire and mini-spirometer to help detect asthma and COPD in real-life clinical practice: a cross-sectional study. NPJ Prim. Care Respir. Med..

